# The Ca^2+^ Sensor Calcineurin B–Like Protein 10 in Plants: Emerging New Crucial Roles for Plant Abiotic Stress Tolerance

**DOI:** 10.3389/fpls.2020.599944

**Published:** 2021-01-15

**Authors:** Felix A. Plasencia, Yanira Estrada, Francisco B. Flores, Ana Ortíz-Atienza, Rafael Lozano, Isabel Egea

**Affiliations:** ^1^Department of Stress Biology and Plant Pathology, Centro de Edafologia y Biologia Aplicada del Segura (CEBAS), Consejo Superior de Investigaciones Científicas (CSIC), Campus Universitario Espinardo, Murcia, Spain; ^2^Centro de Investigación en Biotecnología Agroalimentaria (BITAL), Universidad de Almería, Almería, Spain

**Keywords:** Na^+^ homeostasis, Cacpsdummy2+ homeostasis, Cacpsdummy2+-mediated stress signaling, Cacpsdummy2+-ROS cross-talk, salinity, fruit development, CBL-CIPK

## Abstract

Ca^2+^ is a second messenger that mediates plant responses to abiotic stress; Ca^2+^ signals need to be decoded by Ca^2+^ sensors that translate the signal into physiological, metabolic, and molecular responses. Recent research regarding the Ca^2+^ sensor CALCINEURIN B-LIKE PROTEIN 10 (CBL10) has resulted in important advances in understanding the function of this signaling component during abiotic stress tolerance. Under saline conditions, CBL10 function was initially understood to be linked to regulation of Na^+^ homeostasis, protecting plant shoots from salt stress. During this process, CBL10 interacts with the CBL-interacting protein kinase 24 (CIPK24, SOS2), this interaction being localized at both the plasma and vacuolar (tonoplast) membranes. Interestingly, recent studies have exposed that CBL10 is a regulator not only of Na^+^ homeostasis but also of Ca^2+^ under salt stress, regulating Ca^2+^ fluxes in vacuoles, and also at the plasma membrane. This review summarizes new research regarding functions of CBL10 in plant stress tolerance, predominantly salt stress, as this is the most commonly studied abiotic stress associated with the function of this regulator. Special focus has been placed on some aspects that are still unclear. We also pay particular attention on the proven versatility of CBL10 to activate (in a CIPK-dependent manner) or repress (by direct interaction) downstream targets, in different subcellular locations. These in turn appear to be the link through which CBL10 could be a key master regulator of stress signaling in plants and also a crucial participant in fruit development and quality, as disruption of *CBL10* results in inadequate Ca^2+^ partitioning in plants and fruit. New emerging roles associated with other abiotic stresses in addition to salt stress, such as drought, flooding, and K^+^ deficiency, are also addressed in this review. Finally, we provide an outline of recent advances in identification of potential targets of CBL10, as CBL10/CIPKs complexes and as CBL10 direct interactions. The aim is to showcase new research regarding this master regulator of abiotic stress tolerance that may be essential to the maintenance of crop productivity under abiotic stress. This is particularly pertinent when considering the scenario of a projected increase in extreme environmental conditions due to climate change.

## Introduction: CBL-CIPK, a Versatile Stress Response Mechanism Mediated by Ca^2+^ in Plants

Plants have evolved a complex system to interact with the environment during evolution, which is able to perceive, transduce, and trigger responses to stresses at molecular, cellular, and physiological levels. In this system, calcium ion (Ca^2+^) plays a key role as a second messenger in plant responses to a wide array of environmental stimuli, including abiotic and biotic stress. Advancing in the knowledge of Ca^2+^ -signaling processes is essential in breeding programs for developing crops with enhanced tolerance to adverse environmental conditions. A Ca^2+^ signal is generated when a controlled increase of cytosolic Ca^2+^ occurs. Rise of cytosolic Ca^2+^ is derived either from intracellular stores or from the apoplast. The nature of the stimulus has an influence on the frequency, amplitude, and shape of the Ca^2+^ signal (Dodd et al., 2010). The stimulus generates temporal variations in Ca^2+^ concentration in cytosol, in such a way that the cation encodes precise information, the so-called Ca^2+^ signature. This signature defines the nature and magnitude of the response (Allen et al., 2001; McAinsh and Pittman, 2009). The sources of this Ca^2+^ are the plasma membrane, vacuole, and endoplasmic reticulum (Peiter, 2011). In this picture, activating or deactivating the different components of Ca^2+^ homeostasis (ionic channels or transporters) is requested to generate such Ca^2+^ signal.

For the initiation of the signaling triggered by Ca^2+^ and the activation of cellular and physiological responses, appropriate Ca^2+^ sensors are needed to detect changes in these Ca^2+^ transients. These sensors are Ca^2+^ -binding proteins and are grouped in three major families: calmodulin (CaM) and CaM-like proteins, Ca^2+^ -dependent protein kinases, and calcineurin B–like (CBL) proteins. This last family is constituted by EF-hand Ca^2+^ sensors that present the highest similarity with the regulatory B subunit of phosphatase calcineurin (CNB) protein found in animals. CBLs act as “sensor relays” undergoing conformational changes upon Ca^2+^ binding, allowing for the binding and activation of a family of serine/threonine protein kinases designated as CBL-interacting protein kinases (CIPKs; recently reviewed in Manishankar et al., 2018; Saito and Uozumi, 2020; Tang et al., 2020). CBL/CIPK complexes have been extensively studied by means of the analysis of mutants in *Arabidopsis*, and it has been demonstrated that these complexes mediate Ca^2+^ signals elicited by different environmental stimuli such as cold, abscisic acid (ABA), salinity, osmotic stress, low K^+^ concentration, high pH, and pathogen infection, among others (Kim, 2013). As a result, different CBL/CIPK combinations may engender temporal and spatial specificity in Ca^2+^ signaling and are able to connect diverse stimuli to define cellular responses (Batistič and Kudla, 2012). The intracellular localization of the CBL/CIPK complexes (plasma membrane, nucleus, vacuolar membrane, endoplasmic reticulum, or cytoplasm) is determined by CBLs allowing for spatial specificity in target recognition (D’Angelo et al., 2006; Batistič and Kudla, 2009; Batistič et al., 2010). But binding CIPKs is not the only way of action of CBLs as it has been reported that they may interact in a direct way with target proteins, in a CIPK-independent manner, negatively regulating their activity (Ren et al., 2013; Cho et al., 2016). CALCINEURIN B-LIKE PROTEIN 10 (CBL10), also known as SOS3-LIKE Ca^2+^ BINDING PROTEIN8 (SCABP8), has been recently identified in *Arabidopsis* (Kim et al., 2007; Quan et al., 2007), and recent research shows its function is associated with regulation of ion homeostasis, protecting plant shoots from salt stress. In recent years, new roles in several biotic and abiotic stresses have been attributed to CBL10. In addition, in recent years, progress in establishing the mechanisms of action of CBL10 has been particularly fast, and it has provided a wealth of information on plant cell signaling, which is summarized below.

## The Ca^2+^ Sensor CBL10 Mediates Shoot-Specific Salt Tolerance in Plants

As already said, CBL10 was first identified as a crucial regulator of salt tolerance in *Arabidopsis* (Kim et al., 2007; Quan et al., 2007). The identification and characterization of the first two T-DNA *cbl10* mutants (*Atcbl10* mutants) by these research groups showed that disruption of the *CBL10* gene caused salt hypersensitive phenotypes specifically in shoots, whereas root growth was significantly less affected. In a first phase, salinity reduced chlorophyll content of rosette leaves and inhibited stem elongation of adult mutant plants; later on, the collapsed stems wilted and dried, and finally mutant plants died. The expression profile of the *CBL10* gene detected in *Arabidopsis* wild-type (WT) plants supported such specific shoot salt sensitivity observed in the mutant plants, as transcript amounts were much higher in shoots than in roots (Kim et al., 2007; Quan et al., 2007). Following these pioneering studies, CBL10 has been identified in other species as a crucial regulator in shoot response to salt toxicity. Two CBL10 homologs have been identified in poplar (*Populus trichocarpa*), *PtCBL10A* and *PtCBL10B* (Li et al., 2013; Tang et al., 2014); the first one was ubiquitously expressed at low levels, whereas the second was mainly expressed in the green-aerial tissues. Salt tolerance by maintaining ion homeostasis in shoot tissues is conferred by overexpression of either *PtCBL10A* or *PtCBL10B*, indicating a key role for the gene products in plant response to salt toxicity in shoot. Furthermore, expression of either one in *Arabidopsis cbl10* mutants is able to rescue its salt-sensitive shoot phenotype. Other studies have shown that the heterologous overexpression of homolog *CBL10s* from different species such as tobacco (Dong et al., 2015) enhances the salt tolerance of *Arabidopsis*.

After identification of the *Arabidopsis cbl10* mutants, the only other identified and characterized *cbl10* mutant has been a tomato T-DNA mutant (*Slcbl10* mutant), identified by our research group. We observed that in this tomato mutant *CBL10* disruption induced severe alterations in the shoot apex at different developmental stages (Egea et al., 2018). Thus, in both *in vitro* and *in vivo* salt stress assays, the *Slcbl10* mutant exhibited shoot growth inhibition, hypocotyl thickening, chlorosis at the edge of young leaves, and apical necrosis, and finally plants died because of apical collapse. In agreement with previous studies of *Atcbl10* mutants (Kim et al., 2007; Quan et al., 2007), the *SlCBL10* gene was mainly expressed in aerial tissues of tomato plants. Moreover, expression analysis revealed an upregulation of *SlCBL10* in all vegetative tissues in plants subjected to salinity, especially in upper mature leaves (first fully developed leaves from the apex). Our study demonstrated that the transcriptional activity of *SlCBL10* in this specific tissue is critically involved in the adaptive response of tomato plants to salt stress as it protects shoot apical meristems and growing tissues from physiological damage caused by salinity. In addition, molecular complementation assays proved that *SlCBL10* is orthologous to the *Arabidopsis CBL10* gene and that it rescues the shoot salt-sensitive phenotype of the *Arabidopsis cbl10* mutant.

One of the latest studies on CBL10 has been performed in *Eutrema* (*Eutrema salsugineum*; formerly *Thellungiella halophila*), a halophytic relative of *Arabidopsis*, used as a model for understanding plant adaptation to soil salinity (Monihan et al., 2019). In this study, *CBL10* gene (*EsCBL10*) was found to be duplicated (*EsCBL10a* and *EsCBL10b*), linking this duplication to the high salt tolerance of *Eutrema*, as this species maintains its growth in salt-affected soils where most crop plants are not able to grow. Downregulation, using amiRNAs lines, of either of the duplicated *EsCBL10* genes individually reduced growth in salt stress conditions, hinting that both genes operate in response to salinity. If downregulation of both *EsCBL10* genes is combined, an even greater decrease in growth occurred. This observation suggests that the two genes have either additive effects or different functions, so increasing the number and expanding the activities of this Ca^2+^ sensor via gene duplication confers an adaptive benefit for plant growth in saline soils. While similar levels of *EsCBL10a* transcripts were found throughout the plant (roots, leaves, and flowers), expression profile of *EsCBL10b* was more similar to that of *AtCBL10* in *Arabidopsis*; both transcripts were detected in higher amounts in leaves than in roots. However, when transcript accumulation was compared between leaves and flowers, levels of *EsCBL10b* transcripts were similar in both organs, while transcripts of *AtCBL10* were mainly detected in leaves and only weakly in flowers. Surprisingly, in tomato plants, the highest expression level of *CBL10* was found in flowers (Egea et al., 2018). In fact, tomato *Slcbl10* (Egea et al., 2018) and also *Arabidopsis Atcbl10* (Monihan et al., 2016) displayed an altered flower development under salt stress conditions, which in the case of the tomato mutant led to a significant reduction in fruit yield and quality. Taken together, *CBL10* is a key gene involved in protection of the shoot, of vegetative, and reproductive organs under saline conditions, and this function seems to be conserved among species. However, more studies are needed to elucidate the specific function of CBL10 in flower development as this has been attributed to an indirect effect of the altered function of CBL10 in vegetative tissues.

## CBL10 and Na^+^ Homeostasis: How Does Low Na^+^ Accumulation, Induced by Disruption of CBL10, Provoke Such High Salt Sensitivity in Plants?

The main effect induced by salinity is the toxic effect; this is widely recognized in numerous species to be largely due to Na^+^, as the accumulation of this cation in cytoplasm interferes with multiple metabolic processes (Munns and Tester, 2008). Thus, plants with either less uptake or increased cellular efflux of Na^+^ are generally more tolerant to salinity (Hasegawa, 2013; Maathuis et al., 2014). However, one of the common characteristics of *cbl10* mutants, both in tomato and *Arabidopsis*, is a significantly lower Na^+^ accumulation in shoots under salt stress conditions. Therefore, it is difficult to understand how the salt hypersensitive phenotypes of *cbl10* mutants are associated with low shoot Na^+^ accumulation, when the opposite would be expected.

The mechanism of salinity tolerance in glycophytes is characterized by a favored Na^+^ accumulation in mature leaves and stem, preventing the cation to reach the shoot apex and other growing tissues (Cuartero et al., 2010). In order to explain how *Slcbl10* plants accumulate less Na^+^ but exhibit a salt-hypersensitive phenotype, a precise investigation on Na^+^ partitioning in the tomato mutant was carried out, analyzing separately the young and mature parts of the shoot (Egea et al., 2018). One of the features of *Slcbl10* is its inability to retain Na^+^ in mature tissues so it reaches the shoot apex. This fact matches with the damage observed in apex of the mutant and strongly supports the hypothesis that *SlCBL10* gene is involved in shoot apex protection in salt stress conditions. Therefore, the fact that a low accumulation of Na^+^ causes a greater salt sensitivity in *cbl10* mutants is due to an altered distribution of Na^+^ within the shoot.

There is controversy regarding the mechanism by which CBL10 is involved in the regulation of Na^+^ homeostasis. Whereas some authors place CBL10 at the plasma membrane as a member of the Salt Overly Sensitive (SOS) pathway, involved in Na^+^ extrusion to apoplast, others have localized CBL10 at the tonoplast, suggesting that it could be involved in the activation of a still unknown Na^+^ transporter, compartmentalizing excess of Na^+^ into the vacuole. Both transport processes are recognized as tolerance mechanisms evolved by plants to cope with Na^+^ toxicity by avoiding its accumulation in cytoplasm (Munns and Tester, 2008).

### Is CBL10 a Member of the SOS Pathway at the Plasma Membrane?

**FIGURE 1:**
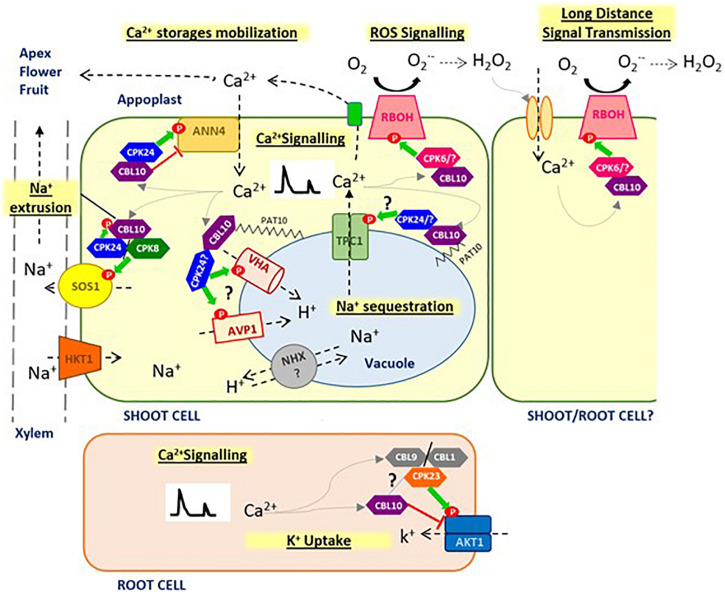
Proposed model for CBL10 function. CBL10 functions as a crucial regulator in Na^+^ homeostasis under salt stress conditions, interacting with CIPK24 (SOS2) and CIPK8, and activating SOS1 pathway at plasma membrane for Na^+^ extrusion to apoplast and may be interacting with vacuolar H^+^ pumps (AVP1, VHA) facilitating Na^+^ sequestration into the vacuole. CBL10 also plays a key role in Ca^2+^ homeostasis, which in turn hints at this Ca^2+^ -sensor protein as a key master regulator of stress signaling in plants, involved in generation and fine tuning of Ca^2+^ signatures in the cytoplasm, through the coordination of different Ca^2+^ channels at different cellular localizations, such as plasma membrane (AtANN4) and probably tonoplast (TPC1), as well as in the cross-talk between Ca^2+^ and ROS, through regulation of RBOHs activity, for a rapid long-distance transmission of signals. CBL10 seems also to be involved in mobilizing Ca^2+^ stored in vacuole toward demanding tissues to compensate for Ca^2+^ deficiency caused by salinity and thus allowing development of the meristem and reproductive organs. Finally, functions of CBL10 in root have been associated with K^+^ uptake by negative regulation of AKT1 channel. Green arrows indicate activation by phosphorylation; red line indicates repression by direct interaction; gray arrows indicate activation by Ca^2+^, and question mark indicates hypothesized signaling processes that have not been verified yet.

In the SOS pathway, a Ca^2+^ -binding protein SOS3 (also known as Calcineurin B-Like Protein 4, CBL4) senses the salt-elicited Ca^2+^ signal and then binds to and activates the SOS2 Ser/Thr protein kinase (also known as CBL-Interacting Protein Kinase 24, CIPK24). Later, the SOS2/SOS3 complex phosphorylates and activates the plasma membrane Na^+^/H^+^ antiporter SOS1, which extrudes excess of Na^+^ from the cytosol to extracellular medium (Qiu et al., 2002; Quintero et al., 2002). The first study involving CBL10 in the SOS pathway was carried out by Quan et al. (2007). These authors showed that AtCBL10 interacted with AtCIPK24 both *in vitro* and *in vivo*, and such interaction enhanced the kinase activity of AtSOS2. By transient expression of AtCBL10 fused to green fluorescent protein in onion epidermal cells, they also showed that AtCBL10 recruits AtCIPK24 to the plasma membrane, being the N-terminal hydrophobic domain of AtCBL10 protein crucial for such subcellular localization. Subsequently, this research group supported and complemented this hypothesis by determining that AtCIPK24 also phosphorylates AtCBL10 at Ser-237 and that such phosphorylation induced by salt stress occurs at the membrane level, stabilizing CBL10–CIPK24 interaction and enhancing AtSOS1 activity (Lin et al., 2009; Figure 1). Results from these studies demonstrate that although AtCBL10 exhibited a higher ability for similar functions than AtSOS3, both CBL proteins must be involved in different regulatory functions in the plant response to salt stress in *Arabidopsis*, as *AtSOS3* is expressed only in roots, whereas *AtCBL10* is mainly expressed in shoots. *cbl10* and *sos3 Arabidopsis* mutants display shoot- and root-specific salt sensitivities, respectively, and these should arise, at least partially, from these different spatial expression profiles. The activation by CBL10 of Na^+^/H^+^ antiporter SOS1 at the plasma membrane when the former interacts with CIPK8 instead of CIPK24 has been recently demonstrated (Yin et al., 2019). Thus, it seems that CBL10–CIPK8 complex positively regulates SOS1 activity and that the CBL10–CIPK8-SOS1 pathway is able to efficiently extrude the excess Na^+^ in yeast cells subjected to salinity. In *Arabidopsis* plants exposed to increasing salinity conditions, activation of SOS1 by phosphorylation triggered by interacting CBL10–CIPK8 occurs at the same sites of the C-terminus domain (Ser-1136 and Ser-1138) as for the same reaction produced by CBL10–SOS2 (Figure 1).

In *Eutrema*, CBL10 is also involved in the SOS pathway; the two identified duplicate *EsCBL10* genes complemented the *Atcbl10* mutant, which demonstrates the conservation of the function of CBL10 in these species. Nevertheless, a divergence for the activity of both *Eutrema* proteins was evidenced when assessing the different capacities of EsCBL10a and EsCBL10b to trigger SOS pathway (Monihan et al., 2019). Cross-species complementation assays have allowed to confirm that only *EsCBL10b* is able to enhance activation of the SOS pathway; furthermore, in yeast, the interaction of two-hybrid assays of this regulator was observed with AtSOS2 at a similar level as AtCBL10. EsCBL10a is able to activate the SOS pathway but only weakly and displays little interaction with CIPK24, hinting at a function for EsCBL10a not performed by AtCBL10 or EsCBL10b. Experiments with chimeric proteins revealed that changes in the N-terminal domain are responsible for the role of EsCBL10b in the SOS pathway and of EsCBL10a in an alternative pathway, and these changes may be involved in the different localizations of the proteins and even in different CIPK interactions.

Despite all these studies that involve CBL10 with the SOS pathway, the *in vivo* functional relationship of SOS and CBL10–regulated processes remains unclear. In fact, controversial results have been obtained when Na^+^ concentration and distribution were analyzed in plants showing impairment in the function of CBL10. Thus, while *sos* mutants accumulate more Na^+^ than WT plants, the opposite is observed in *cbl10* mutants; i.e., there is less accumulation of Na^+^ in the plant (Kim et al., 2007; Quan et al., 2007; Egea et al., 2018). Furthermore, when any *SOS* mutation (*sos3* or *sos1*) is combined with the *cbl10* mutation, a reduced Na^+^ accumulation occurs compared with the simple *sos1* mutation under salinity, despite the fact that the double mutants exhibited similar salt-sensitive phenotypes to *sos1* at the whole plant level (Yang et al., 2019). If the function of CBL10 only resulted in the extrusion of Na^+^ out of the cell via the SOS pathway, a greater accumulation of Na^+^ in the plant would be expected, as occurs in any of the *sos* mutants, but not the opposite, suggesting that CBL10 regulates other transport processes besides that involved in the SOS pathway.

### Does CBL10 Work by Compartmentalizing Excess Na^+^ Into the Vacuole?

In addition to extruding Na^+^ out of the cell, plants have evolved other tolerance mechanisms to cope with salt stress such as compartmentalizing excess of Na^+^ into the vacuole (Munns and Tester, 2008). The first study involving CBL10 in this strategy suggested that the high salt sensitivity of the *Atcbl10* mutant was due to alteration in Na^+^ vacuolar sequestration in leaves (Kim et al., 2007). Thus, when storage in vacuoles was lower, the amount of saline ions that would penetrate plant cells before these die of salt toxicity was lesser, which would explain why the *Atcbl10* mutant contains less Na^+^ than the WT, although the former is much more salt-sensitive. SOS2-CBL10 was localized at the tonoplast instead of the plasma membrane in the study of Kim et al. (2007); moreover, Waadt et al. (2008) confirmed this subcellular localization by bimolecular fluorescence complementation. In other species such as *Populus*, the interaction of CBL10 and CIPK24 was also observed at the vacuolar level (Tang et al., 2014).

Post-translational modification studies on the CBL10 protein support such subcellular localization at the tonoplast: one cysteine residue of CBL10 at the N-terminus, Cys-38 (C38), was predicted to be an S-acylation site by CSS-Palm prediction software (Ren et al., 2008). Chai et al. (2019) proposed S-Acyl Transferase 10 (PAT10) protein as the enzyme responsible for CBL10 association with tonoplast through S-acylation of CBL10 at C38 (Figure 1). Thus, the point mutation of C38S in CBL10 determined an intracellular localization mainly situated in cytoplasm (Chai et al., 2019). However, the expression of the point-mutated version CBL10^C38S^ was at least in part able to enhance growth of *cbl10* mutant, suggesting that the role of CBL10 in salinity responses might be involved at subcellular locations other than tonoplast. Given that S-acylation is a reversible process, this modification could be involved in regulation of CBL10 partitioning among different cellular membranes-compartments (plasma membrane, tonoplast, chloroplast membrane, etc.). Nevertheless, a consensus model has not still been found in order to predict how S-acylation could modulate the traffic of a specific protein among diverse cell membranes, although some studies suggest that the degree of S-acylation could play a role in this modulation (Chamberlain and Shipston, 2015). Regarding this aspect, several N-terminal cysteine residues of CBL10 have been predicted to be S-acylation sites (Ren et al., 2008).

Identification of targets of CBL10 at the tonoplast for vacuolar Na^+^ sequestration still remains a challenge. Initial studies suggested that CBL10–CIPK24 complex may regulate vacuolar transporters Na^+^/H^+^ Exchangers (NHXs) for Na^+^ sequestration by phosphorylation. In support of this hypothesis in a study of salt tolerance in soybean (*Glycine max*), it was observed that the salt-tolerant variety S111-9 exhibited much higher Na^+^ accumulation in the vacuole than the salt-sensitive variety Melros, which was associated with a higher expression of the *CBL10*, *CIPK24*, and *NHX* genes (He et al., 2015). However, earlier studies have shown evidence that tonoplast-localized NHX is mainly involved in K^+^ storage, with little bearing on Na^+^ sequestration (Bassil et al., 2011; Barragán et al., 2012; Andres et al., 2014). In addition, recent genetic evidence in *Arabidopsis* implies that vacuole-localized antiporters NHX1-4 exhibit Na^+^-transport activities, but their contribution to vacuolar Na^+^ compartmentation is small, as the quadruple knockout mutant *nhx1/2/3/4* does not show higher susceptibility to NaCl than WT. Furthermore, vacuoles isolated from the quadruple mutant still retain Na^+^ uptake capability, which hints at the existence in tonoplast of NHX-independent Na^+^ transporters (Bassil et al., 2019).

Characterization of the *Slcbl10* tomato mutant showed that, in response to salinity, the expression pattern of main genes responsible for Na^+^ distribution and compartmentation *SlNHX4*, *SlSOS1*, and *SlSOS2*, *SlHKT1;2* (*HIGH-AFFINITY K^+^ TRANSPORTER 2*) was altered (Egea et al., 2018). In addition, the vacuolar H^+^ pumps *SlAVP1* (*H^+^-PYROPHOSPHATASE, AVP1*) and *SlVHA-A1* (*VACUOLAR H^+^-ATPases, V-ATPase*) gene expressions are altered in the upper leaves close to the apical tissues of the tomato mutant. A severe downregulation of both proton vacuolar pumps (*SlAVP1* and *SlVHA-A1*) responsible for generating the required driving force for Na^+^ compartmentation into the vacuole, as well as moderated downregulation of the Na^+^/H^+^ antiporter *NHX4*, was observed in the*Slcbl10* mutant, revealing that loss of function of *SlCBL10* impaired the ability of tomato leaves to sequester Na^+^ into vacuoles. An upregulation of *SlSOS1* and a severe downregulation of *SlHKT1;2* were also observed in mutant leaves, indicating a higher Na^+^ extrusion from the cytoplasm to the apoplast and a lower Na^+^ influx into the cells, respectively, avoiding in such a way the excessive accumulation of Na^+^ in cytoplasm as a consequence of impaired vacuolar Na^+^ storage. Such an expression pattern resulted in a reduced upload of Na^+^ from the xylem, allowing toxic ions to reach the shoot apex, inducing its collapse and subsequent plant death (Egea et al., 2018). These findings reveal that *SlCBL10* is required for the regulation of Na^+^ homeostasis through the activity of gene products involved in the compartmentalization of Na^+^ into vacuoles (Figure 1); this is in agreement with the first hypothesis postulated by Kim et al. (2007), and it is supported by several studies that have established the subcellular localization of CBL10–SOS2 at the tonoplast (Waadt et al., 2008; Tang et al., 2014). Although more studies are necessary to achieve a definitive conclusion, our results revealed two vacuolar H^+^ pumps, *SlAVP1* and *SlVHA-A1*, as potential targets of *SlCBL10* in tomato plants (Egea et al., 2018; Figure 1). In this sense, Batelli et al. (2007) indicated that SOS2 is able to interact and activate the vacuolar H^+^ pump V-ATPase. To drive the compartmentalization of Na^+^ in vacuole mediated by vacuolar Na^+^/H^+^ antiporters, an electrochemical gradient of H^+^ generated by the vacuolar H^+^ pumps *AVP1* and *V-ATPase* is needed (Maeshima, 2000; Hasegawa, 2013), which could explain the controversy generated in previous studies around vacuolar Na^+^/H^+^ antiporters as targets for the CBL10–CIPK24 complex. However, a study in *Arabidopsis* has shown that the vacuolar proton pump V-ATPase (*VHA-a2*, *VHA-a3*) seems not to be involved, at least directly, in sequestration of Na^+^ in the vacuole. It was observed in the tested experimental conditions that H^+^ pump AVP1 activity is enough to carry on this process (Krebs et al., 2010). Conversely, the isoform localized at the trans-Golgi network-early endosome (*VHA-a1*) seems to play a key role in detoxification of Na^+^, pointing out an important role of the endosomal system in Na^+^ uptake (Krebs et al., 2010). In this regard, it would be interesting to verify if a subcellular localization of CBL10 at the endoplasmic reticulum and Golgi-endosomal occurs, as S-acylation also confers ability to attach to the membranes confining the ER and Golgi compartments (Chamberlain and Shipston, 2015).

In summary, *CBL10* plays a key physiological role in protecting different tissues and organs against salt stress, which requires a proper distribution of Na^+^ across tissues and organs, and transport of Na^+^ from the cytosol to vacuoles. A possibility could be, at least for tomato, that participation of CBL10 in both Na^+^ transport processes could be tissue-dependent. Thus, in young growing tissues, which are little vacuolated, CBL10 could be mainly active at the plasma membrane, involving Na^+^ extrusion out of the cell, which would explain why the loss of function of CBL10 in tomato causes a greater accumulation of Na^+^ in these tissues. However, in cells of mature leaves, equipped with giant vacuoles, CBL10 could be mainly associated with the tonoplast, participating in sequestration of Na^+^ in the vacuole, and hence, the loss of its function causes a lower accumulation of Na^+^ (Egea et al., 2018). Nevertheless, in addition to both membranes, plasma membrane, and tonoplast, CBL10 has also been associated with other subcellular locations. Translocon of the Outer Membrane of the Chloroplasts 34 (TOC34) was identified as a novel interacting partner of CBL10 occurring at the outer membrane of chloroplasts (Cho et al., 2016), an evidence of the ability of CBL10 to relay Ca^2+^ signals in more diverse subcellular locations than it was currently known.

## A New Identified Role of CBL10 in Ca^2+^ Homeostasis Regulation Under Stress Conditions

Initial studies aimed at the identification of the functional role of CBL10 during salt tolerance were focused on Na^+^ homeostasis, although changes in Ca^2+^ contents were also observed in plants where *CBL10* was disrupted. First, in *cbl10* mutants of *Arabidopsis*, a higher Na^+^ and lower Ca^2+^ accumulation in flowers was detected under salt stress conditions, and the research performed reached the conclusion that *CBL10* is crucial for reproductive development (Monihan et al., 2016). These authors also determined that CBL10 function in reproductive development is independent of CIPK24 and SOS1, as loss of CIPK24 activity had no effect on seed development; furthermore, salt stress affected reproductive development in different ways in *Arabidopsis sos1* and *cbl10* mutants. Thus, it was proposed that CBL10 might interact with another CIPK in flowers to regulate Na^+^ levels during reproductive development, or alternatively, CBL10 could interact with a protein that does not belong to the CIPK family. However, no function in direct regulation of Ca^2+^ homeostasis in plants was attributed to CBL10 in the mentioned study (Monihan et al., 2016).

More recently, evidence from the research on *Slcbl10* tomato mutant implicated CBL10 in cellular functions involved not only in Na^+^ but also in Ca^2+^ homeostasis (Egea et al., 2018). Taking into account that Ca^2+^ is normally compartmentalized within vacuoles, mainly in stems and upper leaves close to apical tissues, the mobilization of Ca^2+^ vacuolar reservoirs by plants subjected to salt stress is essential for growth continuance of sink organs such as juvenile tissues and fruits (Bonales-Alatorre et al., 2013; Zhai et al., 2015). Results showed that Ca^2+^ content in stems and upper mature leaves was reduced by salinity in WT but not in *Slcbl10* mutant, which suggests that the mutant encountered difficulties in mobilizing Ca^2+^ reservoirs (Egea et al., 2018). Moreover, by means of reciprocal grafting assays between WT and *Slcbl10*, we demonstrated that it was retention of Ca^2+^ in mature tissues and not a higher transport of it from root to shoot the cause of the higher Ca^2+^ content found in *Slcbl10* mutant organs under salinity. If that is the case, retention of Ca^2+^ in vacuolated upper mature leaves of *Slcbl10* might prevent the ion reaching developing tissues and fruits in the required concentration. Blossom end rot (BER) is the most known symptom of Ca^2+^ deficiency disorders in tomato fruit (de Freitas et al., 2014; Zhai et al., 2015), and we observed a very high BER incidence in fruits of *Slcbl10* mutants (Egea et al., 2018). Poor plant development, inhibited growth of the apical meristem, smaller leaves that showed evident chlorosis at their edges, and thickened petioles and stems, all of them phenotypical changes induced by Ca^2+^ deficiency, were observed during long-term assays in salt-treated tomato mutant plants. In conclusion, the retention of Ca^2+^ in *Slcbl10* upper mature leaves causes an inefficient supply of Ca^2+^ to other demanding organs, as it has been observed in shoot apices and flowers, severely limiting their development under saline conditions. This fact, together with the higher Na^+^ accumulation observed in mutant shoot apex and flowers, indicates that *SlCBL10* gene participates in the maintenance of an adequate low Na^+^/Ca^2+^ ratio in tomato apex and flowers under saline conditions. Certainly the function of *CBL10* in controlling Na^+^ homeostasis has been previously demonstrated in *Arabidopsis* and other species, but the analysis of *Slcbl10* tomato mutants has emphasized this role of *CBL10* in salt tolerance. However, it is of even greater interest that this study presented evidence of *CBL10* involvement in regulation of Ca^2+^ homeostasis (Egea et al., 2018).

The expression profiles of key genes involved in Ca^2+^ fluxes such as *Cation Exhanger Transporter 1* (*CAX1*), *AVP1*, and *VHA-A1*, required for Ca^2+^ compartmentalization into the vacuole (Cheng et al., 2003; Pittman et al., 2009), and vacuolar ion channel *Two Pore Channel1* (*TPC1*) involved in Ca^2+^ release from vacuoles (Hedrich and Marten, 2011), were comparatively analyzed in *Slcbl10* mutants and WT tomato plants (Egea et al., 2018). Results hinted at the role that *SlCBl10* could play in regulating TPC1 activity, suggesting that the CBL10–CIPK24 complex could directly regulate the TPC1 channel aperture through phosphorylation. In support of this hypothesis, the regulation of TPC1 channel has been proposed to be fulfilled by phosphorylation (Kintzer and Stroud, 2016). Nevertheless, CBL10 could also play an indirect role in regulating this TPC1 activity mediating the acidification of vacuoles through the regulation of vacuolar H^+^ pumps, as low pH is required for aperture of the channel (Kintzer and Stroud, 2016). In fact, both SlAVP1 and SlV-ATPase were also identified in this study as potential targets for SlCBL10 (Egea et al., 2018). Interestingly, the temporal expression profile induced by salt treatment of these four genes in tomato is basically similar, a result that supports the idea of a direct relationship among the functions of these gene products in response to salinity in tomato (Egea et al., 2018).

TPC1 channel, in addition to its function as a calcium-release channel, is also a permeable channel for K^+^ and Na^+^ ions (Hedrich et al., 2018). In addition, Ca^2+^ and Na^+^ ions seem to play opposite roles in the gates operation of TPC1 channel. Thus, when luminal Ca^2+^ concentration increases, activation of the channel is inhibited, whereas the presence of high Na^+^ concentration in lumen seems to alleviate the inhibitory effect of Ca^2+^ (Hedrich et al., 2018). Nevertheless, very little is known up to now regarding the regulation of this channel in salt stress conditions. If CBL10 is implicated in its regulation, this could explain the perturbations found both in Ca^2+^ and Na^+^ homeostasis in *Slcbl10* tomato mutant. On the other hand, the correct activity of the vacuolar H^+^ pumps is also essential for both Ca^2+^ and Na^+^ vacuolar fluxes, providing the necessary driving force to their transport against their electrochemical gradient, which is in accordance with the observed alteration in the distribution of these ions in *Slcbl10* mutants under salt stress (Figure 1). In support of these hypotheses, yeast two-hybrid assay has allowed to demonstrate that SOS2 directly interacted with V-ATPase regulatory subunits B1 and B2 in *Arabidopsis* (Batelli et al., 2007). The premise that Ca^2+^ is also a critical messenger in signal transduction coordinating numerous responses to environmental challenges (Kudla et al., 2018) remarks the fact that the role of CBL10 in plant stress signaling through its participation in vacuolar Ca^2+^ release to the cytoplasm remains an exciting perspective to be investigated.

## CBL10 Potential Key Role During Stress Signaling in Plants

As said, Ca^2+^ is implicated in the fine regulation of responses to developmental processes and environmental perturbations such as biotic and abiotic stresses acting as a key second messenger. Ca^2+^ accumulation events in the cytosol are featured by specific temporal and spatial profiles, and these diverge in amplitude, frequency, and duration (Steinhorst and Kudla, 2013). Spatially defined Ca^2+^ signals are produced because of the slow diffusion of Ca^2+^ in the cytoplasm, combined with a tight regulation of its release from and uptake into the apoplast or diverse intracellular stores. These Ca^2+^ signals encode information about specific stimuli, such as a salt stress signal that is presented to the cell as a particular Ca^2+^ signature. Among intracellular stores, the vacuole is the one by far able to accumulate more remobilizable Ca^2+^ in most plant cells. Total and free Ca^2+^ contents in the vacuole change depending on plant species, cell type, and environmental conditions, and these probably have an impact on vacuolar function and release of vacuolar Ca^2+^ (Peiter, 2011). The only vacuolar Ca^2+^ -permeable channel cloned to date is the slow vacuolar channel TPC1. The involvement of TPC1 in activating-expanding the Ca^2+^ wave in the cytoplasm has been reported, but the exact role of this channel in mediation of stress-induced Ca^2+^ changes and the identification of regulatory mechanisms acting to modulate TPC1 channel gating remains to be elucidated (Evans et al., 2016). Results from analysis of *Slcbl10* tomato mutant suggest that CBL10 could be directly or indirectly linked to TPC1 channel regulation (Egea et al., 2018) and therefore with vacuolar Ca^2+^ release to the cytoplasm with a signaling function (Figure 1).

What is more, recent studies have demonstrated that plant systemic signaling is propped up by a ROS-assisted Ca^2+^ -induced Ca^2+^ -release mechanism involving ROS production by Respiratory Burst Oxidase Homolog proteins (RBOHs), a family of membrane-bound NADPH-oxidase enzymes, and Ca^2+^ release for this process depends on activity of the vacuolar channel TPC1 (Choi et al., 2017). This system established a direct connection between ROS production and Ca^2+^ signaling pathways, and it enables the cell-to-cell communication as a rapid long-distance transmission of signals in plants. In the tomato plant, CBL10, in addition to its possible direct or indirect role in regulating TPC1 channel, has been linked with activation of a plasma membrane RBOH involved in ROS production (Figure 1) in response to biotic stress (de la Torre et al., 2013). CBL10 interacts and recruits CIPK6 to the plasma membrane, where the complex CBL10–CIPK6 activates by phosphorylation of the RBOHB protein contributing to ROS generation during the effector-triggered immunity occurring in the interaction of *Pseudomonas syringae* pv. tomato DC3000 and *Nicotiana benthamiana*. In *Arabidopsis*, AtCBL10 has also been described as a signaling component for ROS production induced by brassinosteroids under salt stress conditions (Kang and Nam, 2016), but if this function is mediated in a CIPK-dependent manner, activating an RBOH, has not been determined. Further studies are needed to confirm if CBL10 could be a key link coordinating TPC1 and RBOH in the cross-talk between Ca^2+^ and ROS for stress signaling in plants.

Others studies hint at a relationship between CBL10 and the generation and fine-tuning of specific Ca^2+^ signatures in cytoplasm under stress conditions. Thus, in rice, it has been found that CBL10 is involved in Ca^2+^ cytoplasmic rise in response to hypoxia as a consequence of flooding (Ye et al., 2018). Analysis of *OsCBL10* promoter sequences of eight cultivars of rice differing in their tolerance to flooding at the germination stage revealed variations that might contribute to their divergent tolerance. The flood-tolerant rice cultivars showed a steady low expression of *OsCBL10* and lower Ca^2+^ influx to the cytoplasm, in comparison with flood-sensitive cultivars. In agreement with this observation, *OsCBL10* overexpression lines, with enhanced cytoplasmic Ca^2+^ flux in comparison to WT, were more sensitive to flooding and hypoxia during rice germination. A study in *Arabidopsis* also determined that CBL10, in tandem with CIPK24, induced and mediated the fine-tuning of an AtANNEXIN4 (AtANN4)–dependent Ca^2+^ signature under salt stress (Ma et al., 2019). AtANNs constitute a family of Ca^2+^ -dependent membrane-binding proteins that mediate salt-induced influx of Ca^2+^ contributing to the increase of cytoplasmic Ca^2+^ with important effects on stress tolerance. AtANN4 mediates salt stress–induced Ca^2+^ transients in plants subjected to salinity, leading to a speedy increment of cytosolic Ca^2+^ levels and resulting in SOS pathway activation. CBL10 promotes phosphorylation of AtANN4 mediated by CIPK24 and this further enhances the interaction of AtANN4 and CBL10. Such direct interaction represses AtANN4-mediated Ca^2+^ transients that generate a salt-specific Ca^2+^ signal (Figure 1). In fact, higher increases in cytoplasmic Ca^2+^ in *sos2* and *cbl10* mutants than in WT plants have been determined, revealing that both CIPK24 and CBL10, when targeted at AtANN4, are involved in a negative feedback loop in order to maintain a specific amplitude and frequency of Ca^2+^ concentration in the cytosol. The final result is activation of the SOS pathway at the plasma membrane by a specific Ca^2+^ signature generated in the whole process. Given this demonstrated function of CBL10 in *Arabidopsis*, it would be necessary to prove if changes in the expression of *TPC1* observed in *Slcbl10* tomato mutant could be a consequence rather than the cause of its altered cytoplasmic Ca^2+^ (Egea et al., 2018).

Taken together, these results seem to place CBL10 as a key master regulator of stress signaling in plants. On the one hand, CBL10 is involved in the generation and fine tuning of Ca^2+^ signatures in the cytoplasm that activate different responses depending on the perceived stress, through the coordination of Ca^2+^ influxes from the apoplast and vacuole via regulation of different channels such as AtANN4 (at plasma membrane) and the potential vacuolar channel TPC1. On the other hand, CBL10 might also be crucial in the cross-talk between Ca^2+^ and ROS, through regulation of RBOHs activity, for rapid long-distance transmission of signals (Figure 1).

## Direct Interactions of CBL10 With Repressing Functions

CBL10 exerts its role as repressor by direct interaction in other targets, aside from AtANN4. One of these other targets is the K^+^ channel AKT1 at the plasma membrane of root cells in *Arabidopsis*, involved in K^+^ uptake (Ren et al., 2013). AKT1 activity is induced by the complex that protein kinase CIPK23 forms with two CBL proteins, CBL1/CBL9 (Li et al., 2006; Xu et al., 2006). Ren et al. (2013) showed that CBL10 can directly interact with AKT1, impairing AKT1-mediated inward K^+^ currents. In fact, overexpression of *AtCBL10* (CBL10–OE) caused a phenotype with a degree of sensitiveness as high as that of *akt1* mutant under low K^+^ conditions, with lower K^+^ contents in both CBL10–OE lines and *akt1* mutant plants than in WT. This study concluded that a competition exists between CBL10 and CIPK23 for binding AKT1, negatively modulating its activity in *Arabidopsis* (Figure 1). However, another study carried out in cotton has shown that the homolog CBL10 in this species is also able to interact with CIPK23 (Lu et al., 2017). Taking into account these results and the precedents in the case of regulation of ATANN4 activity, it remains to be verified whether CBL10 could participate in both processes, the activation of the K^+^ channel via its interaction with CIPK23 and the repression of it by direct interaction, modulating K^+^ flux in roots. In *Eutrema*, CBL10 was also related with regulation of K^+^ homeostasis in roots, in particular EsCBL10a isoform, suggesting this could interact with AtCIPK6 and/or AtCIPK16 proteins to regulate K^+^ levels in roots (Monihan et al., 2019).

Another direct interaction of CBL10 with a repressive function is the one described for TOC34 protein (Cho et al., 2016). TOC34 is a member of the TOC (translocon of the outer membrane of the chloroplasts) complex with GTPase activity, regulating protein importation into chloroplasts. The *in vivo* interaction of CBL10 and TOC34 that takes place at the outer membrane of chloroplasts has been observed. Additionally, the physical association of CBL10 with TOC34 has been determined *in vitro*, resulting in a significant diminution of the TOC34 GTPase activity. Taking into account the critical functions of chloroplasts in photosynthesis and other metabolic processes, the integration of this organelle within the Ca^2+^ -signaling network should be considered, and its regulation is certainly needed for adaptation to environmental changes.

Given the demonstrated ability of CBL10 to activate (through a CIPK-dependent manner) or repress (by direct interaction) the activity of an array of targets, it seems logical that loss of function of CBL10 may have positive or negative effects against different types of stress. Thus, the role of CBL10 as a positive regulator of salt stress is well-established (Kim et al., 2007; Quan et al., 2007; Lin et al., 2009; Tang et al., 2014; Kang and Nam, 2016; Egea et al., 2018; Ma et al., 2019; Monihan et al., 2019; Yang et al., 2019), and it appears to display a positive role in pathogen infection (de la Torre et al., 2013). However, opposite effects have been described for other stresses, such as flooding during rice germination in the mentioned research work of Ye et al. (2018), where a lower constitutive *OsCBL10* gene expression was linked with a higher hypoxia tolerance, where it was observed that *OsCBL10* overexpression lines exhibited significant susceptibility to flooding and hypoxia during their germination. Also, under K^+^ deficit, overexpression of *AtCBL10* caused a more sensitive phenotype with lower K^+^ accumulation than in WT plants (Ren et al., 2013). Another stress described in the literature in which CBL10 seems to be implied as a negative regulator is drought. Thus, loss of function of CBL10 in *Arabidopsis* improved the resumed growth of plants after a period of dehydration compared with WT, whereas CBL10–OE plants did not recover such growth (Kang and Nam, 2016). The stomatal sensitivity of *Atcbl10* mutants and CBL10–OE plants to ABA was similar to that of WT plants, and the analysis of the expression of drought marker genes (*COR15A*, *RD29A*, and *DREB2A*) did not clarify the drought tolerance mechanism observed in the mutant, hinting at the fact that the drought tolerant *Atcbl10* phenotype is caused by specific but still unknown processes. Further studies in this direction should be carried out to clarify the relationship between CBL10 and drought stress as well as with other abiotic stressors that have not been studied to date, such as the high or cold temperatures that now commonly occur in crop plantations as a consequence of climate change.

## Concluding Remarks and Future Prospects

**FIGURE 2:**
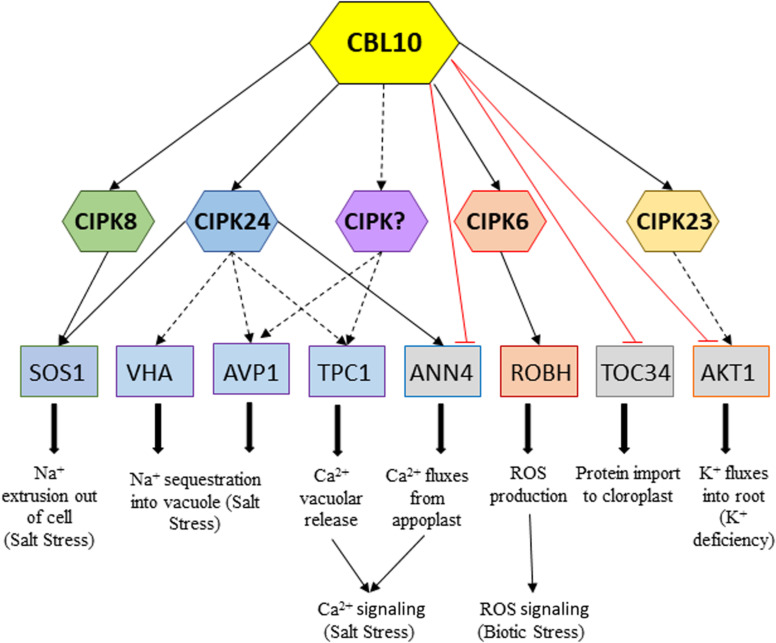
Network of downstream CBL10–CIPKs interactions and CBL10 direct interactions activating or repressing targets in response to different stresses, respectively. Continuous black arrows indicate proved interaction and/or activation by phosphorylation; dotted black arrows indicate interactions still remain to be demonstrated, and red lines indicate demonstrated repressive interactions.

In recent years, research on the Ca^2+^ sensor CBL10 has yielded important advances in the understanding of functions of this signaling component in different facets of plant development and stress tolerance. Altogether, results from these studies seem to point to CBL10 as a key master regulator of stress in plants. This hypothesis is supported by the demonstrated ability of CBL10 to interact with diverse CIPKs, downward activating specific targets, as well as through the demonstrated possibility of its direct interaction with targets repressing their activity, in such a way that opens the possibility of modulating the response to a particular stressor (Figure 2). The role of CBL10 has mainly been studied in the salt stress response, although recent research is expanding its role to examine other stresses, such as biotic (only studied in tomato up to date) and other abiotic ones such as drought, flooding, and low K^+^. To further advance in our understanding of the regulatory roles of CBL10 of different stressors, a crucial but challenging aim is to identify new target transporters activated by CBL10–CIPK complexes or repressed by the direct interaction with CBL10.

The function of CBL10 seems to involve an organ-dependent component: in roots, its main function is related to the regulation of K^+^ homeostasis, whereas in shoots it is related to regulation of Na^+^ and Ca^2+^ homeostasis. Regarding its role in Na^+^ homeostasis, there is controversy with respect to the response mechanism triggered by CBL10. On the one hand, it has been demonstrated that CBL10 is able to activate the SOS pathway in the plasma membrane, for Na^+^ extrusion to the apoplast. On the other hand, CBL10 has also been localized in the tonoplast, supporting its role in Na^+^ sequestration in vacuoles. Taken together, these results and the patterns of Na^+^ distribution in plants where CBL10 has been blocked or inhibited, indicate its role in young tissues, where few vacuoles are present, would be Na^+^ extrusion to apoplast, via the SOS pathway, in order to prevent an excessive accumulation that would lead to cessation of plant growth and development. But there are elements of evidence supporting the hypothesis, which still needs to be verified, that CBL10 may participate in Na^+^ sequestration in vacuoles of cells from mature leaves, avoiding the risk that this toxic ion could be transported to and reach too high concentrations in young growing tissues. In this last scenario, identification of CBL10 targets in tonoplast for vacuolar Na^+^ sequestration is therefore a priority in future research.

Regarding Ca^2+^ homeostasis, CBL10 seems to be involved in mobilizing vacuolar Ca^2+^ toward demanding tissues, in order to compensate for Ca^2+^ deficiency caused by salinity and thus allowing the development of the meristem and reproductive organs. Furthermore, it could also be involved in Ca^2+^ flux from vacuoles to cytoplasm with signaling purposes. Within this framework, some tonoplast proteins have been identified as potential targets for CBL10, such as the H^+^ pumps AVP1 and V-ATPase (VHA), and TPC1 channel, although direct regulation by CBL10 in a CIPK-dependent or -independent manner remains to be explored. On the other hand, given the role of CBL10 in Ca^2+^ homeostasis, it would be very interesting to elucidate the possible roles of CBL10 in fruit development, production, and quality, as well as in the incidence of common fruit physiological disorders that have been linked to Ca^2+^ deficiency in fruit (e.g., BER in tomatoes). In fact, Ca^2+^ has an important role in membrane stabilization, water relations, and cell wall properties modification via cross-linking of de-esterified pectins, and these processes may have an important impact in fruit development, physical traits, and disease susceptibility (Hocking et al., 2016). It is even possible that CBL10 might have a role in the postharvest life of fruit. In support of this last hypothesis, a transcriptomic study of oranges stored at room temperature has shown results of an increase in the homolog *CBL10* expression during fruit senescence (Tang et al., 2016). With regard to regulation of Ca^2+^ homeostasis, results from different studies seem to place CBL10 as a key master regulator of stress signaling in plants, involved in the generation and fine tuning of Ca^2+^ signatures in the cytoplasm, through coordination of different Ca^2+^ channels at different cellular locations, such as the plasma membrane (AtANN4) or the tonoplast (TPC1), as well as in the cross-talk between Ca^2+^ and ROS via regulation of RBOH activity, for rapid long-distance signal transmission. However, the direct interaction of CBL10 in the regulation of TPC1 needs to be verified, and identification of specific RBOHs regulated by CBL10 for long-distance signal transmission requires further research.

In conclusion, despite so important advances achieved in recent years, further and deeper studies are required to elucidate the complex roles of CBL10 in key functions during plant development under abiotic stress conditions.

## Author Contributions

FAP and YE wrote the original draft copy. FBF and AO-A revised and edit the manuscript contributing with new ideas. RL and IE conceived the idea of the review article, supervised the project, revised the manuscript, and finished the writing.

## Conflict of Interest

The authors declare that the research was conducted in the absence of any commercial or financial relationships that could be construed as a potential conflict of interest.

## References

[B1] Allen G. J. Chu S. P. Harrington C. L. Schumacher K. Hoffmann T. Tang Y. Y. (2001). A defined range of guard cell calcium oscillation parameters encodes stomatal movements. *Nature* 411 1053–1057. 10.1038/35082575 11429606

[B2] Andres Z. Pérez-Hormaeche J. Leidi E. O. Schlücking K. Steinhorst L. McLachlan D. H. (2014). Control of vacuolar dynamics and regulation of stomatal aperture by tonoplast potassium uptake. *Proc. Natl. Acad. Sci. U.S.A.* 111 E1806–E1814. 10.1073/pnas.1320421111 24733919 PMC4035970

[B3] Barragán V. Leidi E. O. Andrés Z. Rubio L. Luca A. Fernández J. A. (2012). Ion Exchangers NHX1 and NHX2 mediate active potassium uptake into vacuoles to regulate Cell Turgor and Stomatal function in *Arabidopsis*. *Plant Cell* 24 1127–1142. 10.1105/tpc.111.095273 22438021 PMC3336136

[B4] Bassil E. Tajima H. Liang Y. C. Ohto M. A. Ushijima K. Nakano R. (2011). The *Arabidopsis* Na+/H+ Antiporters NHX1 and NHX2 control vacuolar pH and K+ homeostasis to regulate growth, flower development, and reproduction. *Plant Cell* 23 3482–3497. 10.1105/tpc.111.089581 21954467 PMC3203450

[B5] Bassil E. Zhang S. Gong H. Tajima H. Blumwald E. (2019). Cation specificity of vacuolar NHX-Type Cation/H+ antiporters. *Plant Physiol.* 179 616–629. 10.1104/pp.18.01103 30498025 PMC6426403

[B6] Batelli G. Verslues P. E. Agius F. Qiu Q. Fujii H. Pan S. (2007). SOS2 promotes salt tolerance in part by interacting with the vacuolar H+-ATPase and upregulating its transport activity. *Mol. Cell. Biol.* 27 7781–7790. 10.1128/MCB.00430-07 17875927 PMC2169139

[B7] Batistič O. Kudla J. (2009). Plant calcineurin B-like proteins and their interacting protein kinases. *Biochim. Biophys. Acta* 1793 985–992. 10.1016/j.bbamcr.2008.10.006 19022300

[B8] Batistič O. Kudla J. (2012). Analysis of calcium signaling pathways in plants. *Biochim. Biophys. Acta* 1820 1283–1293. 10.1016/j.bbagen.2011.10.012 22061997

[B9] Batistič O. Waadt R. Steinhorst L. Held K. Kudla J. (2010). CBL-mediated targeting of CIPKs facilitates the decoding of calcium signals emanating from distinct cellular stores. *Plant J.* 61 211–222. 10.1111/j.1365-313X.2009.04045.x 19832944

[B10] Bonales-Alatorre E. Pottosin I. Shabala L. Chen Z. H. Zeng F. Jacobsen S. E. (2013). Differential activity of plasma and vacuolar membrane transporters contributes to genotypic differences in salinity tolerance in a halophyte species, *Chenopodium quinoa*. *Int. J. Mol. Sci.* 14 9267–9285. 10.3390/ijms14059267 23629664 PMC3676782

[B11] Chai S. Ge F. R. Zhang Y. Li S. (2019). S-acylation of CBL10/SCaBP8 by PAT10 is crucial for its tonoplast association and function in salt tolerance. *J. Integr. Plant Biol.* 62 718–722. 10.1111/jipb.12864 31441225

[B12] Chamberlain L. H. Shipston M. J. (2015). The physiology of protein S-acylation. *Physiol. Rev.* 95 341–376. 10.1152/physrev.00032.2014 25834228 PMC4551212

[B13] Cheng N. H. Pittman J. K. Barkla B. J. Shigaki T. Hirschi K. D. (2003). The Arabidopsis *cax1* mutant exhibits impaired ion homeostasis, development, and hormonal responses and reveals interplay among vacuolar transporters. *Plant Cell* 15 347–364. 10.1105/tpc.007385 12566577 PMC141206

[B14] Cho J. H. Lee J. H. Park Y. K. Choi M. Kim K. N. (2016). Calcineurin B-like protein CBL10 directly interacts with TOC34 (Translocon of the Outer Membrane of the Chloroplasts) and decreases its GTPase activity in *Arabidopsis*. *Front. Plant Sci.* 7:1911. 10.3389/fpls.2016.01911 28018422 PMC5156837

[B15] Choi W. G. Miller G. Wallace I. Harper J. Mittler R. Gilroy S. (2017). Orchestrating rapid long-distance signaling in plants with Ca2+, ROS and electrical signals. *Plant J.* 90 698–707. 10.1111/tpj.13492 28112437 PMC5677518

[B16] Cuartero J. Bolarin M. C. Moreno V. Pineda B. (2010). “Molecular tools for enhancing salinity tolerance in plants,” in *Molecular Techniques in Crop Improvement*, eds Jain S. M. Brar D. S. (New York, NY: Springer), 373–405. 10.1007/978-90-481-2967-6_16

[B17] D’Angelo C. Weinl S. Batistič O. Pandey G. K. Cheong Y. H. Schültke S. (2006). Alternative complex formation of the Ca2+-regulated protein kinase CIPK1 controls abscisic acid-dependent and independent stress responses in Arabidopsis. *Plant J.* 48 857–872. 10.1111/j.1365-313X.2006.02921 17092313

[B18] de Freitas S. T. McElrone A. J. Shackel K. A. Mitcham E. J. (2014). Calcium partitioning and allocation and blossom-end rot development in tomato plants in response to whole-plant and fruit-specific abscisic acid treatments. *J. Exp. Bot.* 6 235–247. 10.1093/jxb/ert364 24220654 PMC3883292

[B19] de la Torre F. Gutiérrez-Beltrán E. Pareja-Jaime Y. Chakravarthy S. Martin G. B. del Pozo O. (2013). The tomato calcium sensor Cbl10 and its interacting protein kinase Cipk6 define a signaling pathway in plant immunity. *Plant Cell* 25 2748–2764. 10.1105/tpc.113.113530 23903322 PMC3753395

[B20] Dodd A. N. Kudla J. Sanders D. (2010). The language of calcium signaling. *Annu. Rev. Plant Biol.* 61 593–620. 10.1146/annurev-arplant-070109-104628 20192754

[B21] Dong L. Wang Q. Manik S. M. N. Song Y. Shi S. Su Y. (2015). Nicotiana sylvestris calcineurin B-like protein NsylCBL10 enhances salt tolerance in transgenic *Arabidopsis*. *Plant Cell Rep.* 12 2053–2063. 10.1007/s00299-015-1851-4 26318216

[B22] Egea I. Pineda B. Ortíz-Atienza A. Plasencia F. A. Drevensek S. García-Sogo B. (2018). The SlCBL10 Calcineurin B-Like protein ensures plant growth under salt stress by regulating Na+ andCa2+ homeostasis. *Plant Physiol.* 176 1676–1693. 10.1104/pp.17.01605 29229696 PMC5813568

[B23] Evans M. J. Choi W. G. Gilroy S. Morris R. J. (2016). A ROS-assisted calcium wave dependent on the AtRBOHD NADPH oxidase and TPC1 cation channel propagates the systemic response to salt stress. *Plant Physiol.* 171 1771–1784. 10.1104/pp.16.00215 27261066 PMC4936552

[B24] Hasegawa P. M. (2013). Sodium (Na+) homeostasis and salt tolerance of plants. *Environ. Exp. Bot.* 92 19–31. 10.1016/j.envexpbot.2013.03.001

[B25] He Y. Fu J. Yu C. Wang X. Jiang Q. Hong J. (2015). Increasing cyclic electron flow is related to Na+ sequestration into vacuoles for salt tolerance in soybean. *J. Exp. Bot.* 66 6877–6889. 10.1093/jxb/erv392 26276865 PMC4623694

[B26] Hedrich R. Marten I. (2011). TPC1-SV channels gain shape. *Mol. Plant* 4 428–441. 10.1093/mp/ssr017 21459829

[B27] Hedrich R. Mueller T. D. Becker D. Marten I. (2018). Structure and function of TPC1 vacuole SV channel gains shape. *Mol. Plant* 11 764–775. 10.1016/j.molp.2018.03.017 29614320

[B28] Hocking B. Tyerman S. D. Burton R. A. Gilliham M. (2016). Fruit calcium: transport and physiology. *Front. Plant Sci.* 7:569. 10.3389/fpls.2016.00569 27200042 PMC4850500

[B29] Kang H. K. Nam K. H. (2016). Reverse function of ROS-induced CBL10 during salt and drought stress responses. *Plant Sci.* 243 49–55. 10.1016/j.plantsci.2015.11.006 26795150

[B30] Kim B. G. Waadt R. Cheong Y. H. Pandey G. K. Dominguez-Solis J. R. Schültke S. (2007). The calcium sensor CBL10 mediates salt tolerance by regulating ion homeostasis in Arabidopsis. *Plant J.* 52 473–484. 10.1111/j.1365-313X.2007.03249.x 17825054

[B31] Kim K. N. (2013). Stress responses mediated by the CBL calcium sensors in plants. *Plant Biotechnol. Rep.* 7 1–8. 10.1007/s11816-012-0228-1

[B32] Kintzer A. F. Stroud R. M. (2016). Structure, inhibition and regulation of two pore channel TPC1 from *Arabidopsis thaliana*. *Nature* 531 258–262. 10.1038/nature17194 26961658 PMC4863712

[B33] Krebs M. Beyhl D. Görlich E. Al-Rasheid K. A. S. Marten M. Stierhof Y. D. (2010). Arabidopsis V-ATPase activity at the tonoplast is required for efficient nutrient storage but not for sodium accumulation. *Proc. Natl. Acad. Sci. U.S.A.* 107 3251–3256. 10.1073/pnas.0913035107 20133698 PMC2840351

[B34] Kudla J. Becker D. Grill E. Hedrich R. Hippler M. Kummer U. (2018). Advances and current challenges in calcium signaling. *New Phytol.* 218 414–431. 10.1111/nph.14966 29332310

[B35] Li D. D. Xia X. L. Yin W. L. Zhang H. C. (2013). Two poplar calcineurin B-like proteins confer enhanced tolerance to abiotic stresses in transgenic *Arabidopsis thaliana*. *Biol. Plant* 57 70–78. 10.1007/s10535-012-0251-7

[B36] Li L. Kim B. G. Cheong Y. H. Pandey G. K. Luan S. (2006). A Ca2+ signalling pathway regulates a K+ channel for low-K response in Arabidopsis. *Proc. Natl Acad. Sci. U.S.A.* 103 12625–12630. 10.1073/pnas.0605129103 16895985 PMC1567929

[B37] Lin H. Yang Y. Quan R. Mendoza I. Wu Y. Du W. (2009). Phosphorylation of SOS3-LIKE CALCIUM BINDING PROTEIN8 by SOS2 protein kinase stabilizes their protein complex and regulates salt tolerance in *Arabidopsis*. *Plant Cell* 21 1607–1619. 10.1105/tpc.109.066217 19448033 PMC2700523

[B38] Lu T. Zhang G. Sun L. Wang J. Hao F. (2017). Genome-wide identification of CBL family and expression analysis of *CBLs* in response to potassium deficiency in cotton. *PeerJ* 5:e3653. 10.7717/peerj.3653 28828254 PMC5560230

[B39] Ma L. Ye J. Yang Y. Lin H. Yue L. Luo J. (2019). The SOS2-SCaBP8 complex generates and fine-tunes an AtANN4-dependent calcium signature under salt stress. *Dev. Cell* 48 697–709. 10.1016/j.devcel.2019.02.010 30861376

[B40] Maathuis F. J. M. Ahmad A. Patishtan J. (2014). Regulation of Na+ fluxes in plants. *Front. Plant Sci.* 4:467. 10.3389/fpls.2014.00467 25278946 PMC4165222

[B41] Maeshima M. (2000). Vacuolar H+-pyrophosphatase. *Biochim. Biophys. Acta* 1465 37–51. 10.1016/S0005-2736(00)00130-9 10748246

[B42] Manishankar P. Wang N. Köster P. Alatar A. A. Kudla J. (2018). Calcium signaling during salt stress and in the regulation of ion homeostasis. *J. Exp. Bot.* 69 4215–4226. 10.1093/jxb/ery201 29800460

[B43] McAinsh M. R. Pittman J. K. (2009). Shaping the calcium signature. *New Phytol.* 181 275–294. 19121028 10.1111/j.1469-8137.2008.02682.x

[B44] Monihan S. M. Magness C. A. Yadegari R. Smith S. E. Schumaker K. S. (2016). Arabidopsis CALCINEURIN B-LIKE10 functions independently of the SOS pathway during reproductive development in saline conditions. *Plant Physiol.* 171 369–379. 10.1104/pp.16.00334 26979332 PMC4854721

[B45] Monihan S. M. Ryu C. H. Magness C. A. Schumaker K. S. (2019). Linking duplication of a calcium sensor to salt tolerance in *Eutrema salsugineum*. *Plant Physiol.* 179 1176–1192. 10.1104/pp.18.01400 30606887 PMC6393783

[B46] Munns R. Tester M. (2008). Mechanisms of salinity tolerance. *Annu. Rev. Plant Biol.* 59 651–681. 10.1146/annurev.arplant.59.032607.092911 18444910

[B47] Peiter E. (2011). The plant vacuole: emitter and receiver of calcium signals. *Cell Calcium* 50 120–128. 10.1016/j.ceca.2011.02.002 21376393

[B48] Pittman J. K. Edmond C. Sunderland P. A. Bray C. M. (2009). A cation regulated and proton gradient-dependent cation transporter from *Chlamydomonas reinhardtii* has a role in calcium and sodium homeostasis. *J. Biol. Chem.* 284 525–533. 10.1074/jbc.M807173200 19001368

[B49] Qiu Q. S. Guo Y. Dietrich M. A. Schumaker K. S. Zhu J. K. (2002). Regulation of SOS1, a plasma membrane Na+/H+ exchanger in *Arabidopsis thaliana*, by SOS2 and SOS3. *Proc. Natl. Acad. Sci. U.S.A.* 99 8436–8441. 10.1073/pnas.122224699 12034882 PMC123085

[B50] Quan R. Lin H. Mendoza I. Zhang Y. Cao W. Yang Y. (2007). SCABP8/CBL10, a putative calcium sensor, interacts with the protein kinase SOS2 to protect Arabidopsis shoots from salt stress. *Plant Cell* 19 1415–1431. 10.1105/tpc.106.042291 17449811 PMC1913747

[B51] Quintero F. J. Ohta M. Shi H. Zhu J. K. Pardo J. M. (2002). Reconstitution in yeast of the *Arabidopsis* SOS signaling pathway for Na+ homeostasis. *Proc. Natl. Acad. Sci. U.S.A.* 99 9061–9066. 10.1073/pnas.132092099 12070350 PMC124423

[B52] Ren J. Wen L. Gao X. Jin C. Xue Y. Yao X. (2008). CSS-Palm 2.0: an updated software for palmitoylation sites prediction. *Protein Eng. Des. Sel.* 21 639–644. 10.1093/protein/gzn039 18753194 PMC2569006

[B53] Ren X. L. Qi G. N. Feng H. Q. Zhao S. Zhao S. S. Wang Y. (2013). Calcineurin B-like protein CBL10 directly interacts with AKT1 and modulates K+ homeostasis in *Arabidopsis*. *Plant J.* 74 258–266. 10.1111/tpj.12123 23331977

[B54] Saito S. Uozumi N. (2020). Calcium-regulated phosphorylation systems controlling uptake and balance of plant nutrients. *Front. Plant Sci.* 11:44. 10.3389/fpls.2020.00044 32117382 PMC7026023

[B55] Steinhorst L. Kudla J. (2013). Calcium and reactive oxygen species rule the waves of signaling. *Plant Physiol.* 163 471–485. 10.1104/pp.113.222950 23898042 PMC3793029

[B56] Tang N. Deng W. Hu N. Chen N. Li Z. (2016). Metabolite and transcriptomic analysis reveals metabolic and regulatory features associated with Powell orange pulp deterioration during room temperature and cold storage. *Postharvest Biol. Technol.* 112 75–86. 10.1016/j.postharvbio.2015.10.008

[B57] Tang R. J. Wang C. Li K. Luan S. (2020). The CBL–CIPK calcium signaling network: unified paradigm from 20 years of discoveries. *Trends Plant Sci.* 25 604–617. 10.1016/j.tplants.2020.01.009 32407699

[B58] Tang R. J. Yang Y. Yang L. Liu H. Wang C. T. Yu M. M. (2014). Poplar calcineurin B-like proteins PtCBL10A and PtCBL10B regulate shoot salt tolerance through interaction with PtSOS2 in the vacuolar membrane. *Plant Cell Environ.* 37 573–588. 10.1111/pce.12178 23941462

[B59] Waadt R. Schmidt L. K. Lohse M. Hashimoto K. Bock R. Kudla J. (2008). Multicolor bimolecular fluorescence complementation reveals simultaneous formation of alternative CBL/CIPK complexes in planta. *Plant J.* 56 505–516. 10.1111/j.1365-313X.2008.03612.x 18643980

[B60] Xu J. Li H. D. Chen L. Q. Wang Y. Liu L. L. He L. (2006). A protein kinase, interacting with two calcineurin B-like proteins, regulates K+ transporter AKT1 in Arabidopsis. *Cell* 125 1347–1360. 10.1016/j.cell.2006.06.011 16814720

[B61] Yang Y. Zhang C. Tang R. J. Xu H. X. Lan W. Z. Zhao F. (2019). Calcineurin B-like proteins CBL4 and CBL10 mediate two independent salt tolerance pathways in *Arabidopsis*. *Int. J. Mol. Sci.* 20:2421. 10.3390/ijms20102421 31100786 PMC6566158

[B62] Ye N. H. Wang F. Z. Shi L. Chen M. X. Cao Y. Y. Zhu F. Y. (2018). Natural variation in the promoter of rice calcineurin B-likeprotein10 (OsCBL10) affects flooding tolerance during seed germination among rice subspecies. *Plant J.* 94 612–625. 10.1111/tpj.13881 29495079

[B63] Yin X. Xia Y. Xie Q. Cao Y. Wang Z. Hao G. (2019). The protein kinase complex CBL10–CIPK8–SOS1 functions in *Arabidopsis* to regulate salt tolerance. *J. Exp. Bot.* 71 1801–1814. 10.1093/jxb/erz549 31858132 PMC7242078

[B64] Zhai Y. Yang Q. Hou M. (2015). The effects of saline water drip irrigation on tomato yield, quality, and blossom–end rot incidence: a 3a case study in the south of China. *PLoS One* 10:e0142204. 10.1371/journal.pone.0142204 26540394 PMC4634986

